# CML/RAGE signal induces calcification cascade in diabetes

**DOI:** 10.1186/s13098-016-0196-7

**Published:** 2016-12-28

**Authors:** Zhongqun Wang, Lihua Li, Rui Du, Jinchuan Yan, Naifeng Liu, Wei Yuan, Yicheng Jiang, Suining Xu, Fei Ye, Guoyue Yuan, Baohai Zhang, Peijing Liu

**Affiliations:** 1 Department of Cardiology, Affiliated Hospital of Jiangsu University, 438 Jiefang, Zhenjiang, 212001 China; 2 Department of Pathology, Affiliated Hospital of Jiangsu University, Zhenjiang, 212001 China; 3 Department of Ultrasound, Affiliated Hospital of Jiangsu University, Zhenjiang, 212001 China; 4 Department and Institute of Cardiology, Zhongda Hospital, Southeast University, 87 Dingjiaqiao, Nanjing, 210009 China; 5 Department of Cardiology, Huaian No.1 People’s Hospital, Huaian, 223300 China; 6 Department of Endocrinology, Affiliated Hospital of Jiangsu University, Zhenjiang, 212001 China

**Keywords:** Advanced glycation end-products, p38MAPK, Vascular calcification, Atherosclerosis, Diabetic foot

## Abstract

**Objective:**

Vascular calcification is a significant predictor of coronary heart disease events, stroke, and lower-limb amputation. Advanced glycation end-products (AGEs) play a key role in the development of vascular calcification. However, the role of Nε-carboxymethyl-lysine (CML), a major active ingredient of heterogeneous AGEs, in the development of atherosclerotic calcification in diabetic patients and the underlying mechanism remain unclear. Hence, the role and the mechanism of CML in the transmission pathway of diabetic calcification cascade were investigated in the present study.

**Methods:**

In vivo and in vitro investigations were performed. In study I, 45 diabetic patients hospitalized for above-knee amputation in the Department of Orthopedics, Affiliated Hospital of Jiangsu University were recruited from February 2010 to June 2015. The patients were categorized based on the severity of anterior tibial artery stenosis, which was assessed by color Doppler ultrasound, into mild stenosis (0% < stenosis < 50%, n = 15), moderate stenosis (50 ≤ stenosis < 70%, n = 15), and severe stenosis/occlusion groups (70 ≤ stenosis ≤ 100%, n = 15). In study II, the specific mechanism of CML in the transmission pathway of the diabetic calcification cascade signal was investigated in A7r5 aortic smooth muscle cells under high-lipid, apoptosis-coexisting conditions. ELISA (for serum CML concentration of patients), ultrasound (for plaque size, calcification, blood flow filling, vascular stenosis etc.), H&E staining (for plaque morphology), vonKossa staining (for qualitative analysis of calcification), calcium content assay (for quantitative analysis of calcification), and Western blot analyses of CML, receptor for advanced glycation end products (RAGE), NADPH oxidase 4, phosphorylated p38, core-binding factor α1 (cbfα1), alkaline phosphatase (ALP) and β-actin were then performed.

**Results:**

Morphological analysis revealed extensive calcification lesions in the intima and media of the anterior tibial artery. The extent and area of calcium deposition in the intima significantly increased with disease progression. Interestingly, spotty calcification was predominant in the atherosclerotic plaques of diabetic patients with amputation, and macrocalcification was almost invisible. Pearson correlation analysis revealed that serum CML level exhibited a significant positive correlation with calcium content in the arterial wall (R^2^ = 0.6141, *P* < 0.0001). Semi-quantitative Western blot analysis suggested that the intensity of CML/RAGE signal increased with progression of atherosclerotic calcification in diabetic patients. In subsequent in vitro study, the related pathway was blocked by anti-RAGE antibody, NADPH oxidase inhibitor DPI, p38MAPK inhibitor SB203580, and anti-cbfa1 antibody in a step-wise manner to observe changes in calcium deposition and molecular signals. Results suggested that CML may play a key role in atherosclerotic calcification mainly through the CML/RAGE- reactive oxygen species (ROS)-p38MAPK-cbfα1-ALP pathway.

**Conclusion:**

Spotty calcification was predominant in the atherosclerotic plaques of amputated diabetic patients. CML/RAGE signal may induce the calcification cascade in diabetes via ROS-p38MAPK.

## Background

The International Diabetes Federation reported that the number of diabetic patients worldwide was 387 million in 2014 and is predicted to reach 592 million by 2035 [1]. China has the highest number of people with diabetes, which accounts for about one-third of the total number of diabetic patients worldwide [1]. Patients with diabetes commonly manifest vascular calcification, including intima and medial calcification, which are mainly distributed in the coronary artery and lower extremity vessels, respectively [2, 3]. A multi-center epidemiological study demonstrated that the risk of acute cardiovascular events in patients with calcification score >300 is significantly higher than those in patients with calcification score of 1–100 [4, 5]. Studies showed that the degree of vascular calcification could be the optimal predictor of cardiovascular mortality, stroke incidence, and risk of lower limb amputation in patients with diabetes mellitus [4–8]. Thus, further insights into the mechanism of calcification in diabetic atherosclerosis exhibit theoretical and social significance.

Vascular calcification is an active process in which vascular smooth muscle cells (VSMCs) adopt an osteoblastic phenotype and deposit hydroxyapatite crystals [9]. Our previous studies showed that Nε-carboxymethyl-lysine (CML), a key active ingredient of heterogeneous AGEs, could promote the transdifferentiation of VSMC phenotype and the formation of aortic calcification in diabetic mice [10]. It’s well known that advanced glycation end-products (AGEs) are metabolic products of glucose toxicity and play a significant role in the development of diabetes and its multiple complications. Studies showed that AGEs could accumulate in many tissues and organs, such as skin, kidney, and aorta wall [11, 12]. High baseline AGE levels are significantly associated with plaque progression after adjusting for diabetes mellitus in multivariate logistic regression models [13]. In addition, AGE/the receptor for advanced glycation end-products (RAGE) signal heavily influences both cellular and systemic responses to increase bone matrix proteins through p38MAPK in both hyperglycemic and calcification conditions [14]. The signal in diabetes-mediated vascular calcification was also attributed to increased oxidative stress resulting in the phenotypic switch of VSMCs to osteoblast-like cells in AGEs-induced calcification [14].

Then, what’s the relationship among CML/RAGE, p38MAPK, reactive oxygen species (ROS), core-binding factor α1 (cbfα1, a key transcription factor inducing osteoblastic differentiation) and alkaline phosphatase (ALP, a specific marker of osteoblastic differentiation) in non-crosslink and non-fluorescent CML-induced calcification? There is no accurate answer. And furthermore, the role of CML in the development of atherosclerotic calcification in diabetic patients and the underlying mechanism also remain unclear. Thus, in the present study, the role and the mechanism of CML in the transmission pathway of diabetic calcification cascade signal were investigated through in vivo and in vitro experiments to develop a new perspective for prevention and treatment of atherosclerotic calcification in diabetes mellitus.

## Methods

### Materials

CML was purchased from PolyPeptide Laboratories (San Diego, USA). Oxidized low density lipoprotein (oxLDL) was acquired from Yiyuan Biotechnology (Guangzhou, China). RAW264.7 macrophages and A7r5 aortic vascular smooth muscle cells (VSMC) were obtained from ATCC (USA). CML ELISA kit, calcium assay, and ALP activity kits were provided by Nanjing Jiancheng Bioengineering Institute (Nanjing, China). von Kossa staining kit was acquired from Shunbai Biologicals Inc. (Shanghai, China). SB203580 (p38MAPK inhibitor) and DPI (NADPH oxidase inhibitor) were obtained from Sigma-Aldrich Co. LLC (USA). Anti-Nε-carboxymethyl-Lysine antibody was provided by Abcam (UK). The antibodies against RAGE, NADPH oxidase 4 (Nox4), cbfα1, P-p38MAPK, ALP, β-actin, and all secondary antibodies were provided by Santa Cruz (USA). All other chemicals and reagents were of analytical grade.

### Human studies

Type 2 diabetic patients hospitalized for above-knee amputation in the Department of Orthopedics, Affiliated Hospital of Jiangsu University (Zhenjiang, China) were recruited from February 2010 to June 2015 (n = 45). All patients were treated with standard insulin therapy to control blood glucose. Other comprehensive treatments were used to control local infection, improve blood circulation of lower limbs, supply neurotrophic drugs, and provide debridement/dressing. According to the guidelines for prevention and treatment of type 2 diabetes in China (2013 edition), major amputations are performed in the foot of diabetic patients, whose lower extremities are ineligible for revascularization because of severe ischemia, necrosis, and infection and whose wounds could not heal after circulation improvement, local debridement, or minor amputation (amputation below the ankle joint).

Data regarding age, sex, smoking, duration of diabetes mellitus, hypertension status, fasting plasma glucose (FPG), glycated hemoglobin (HbA1c), lipid profile [total cholesterol, fasting triglycerides, low-density lipoprotein (LDL)-cholesterol, high-density lipoprotein (HDL)-cholesterol], blood urea nitrogen (BUN) and serum creatinine (SCr) were collected before the amputation. All patients were divided into three groups based on the severity of anterior tibial artery stenosis, which was assessed by color Doppler ultrasound: mild stenosis group (0% < stenosis < 50%, n = 15), moderate stenosis group (50 ≤ stenosis < 70%, n = 15), and severe stenosis/occlusion group (70 ≤ stenosis ≤ 100%, n = 15). Written informed consents were obtained from all patients. This study was approved by the Ethical Committee of Jiangsu University and conducted in agreement with the institutional guidelines.

### Detection of lower extremity arteries by color Doppler ultrasound

Related indices of lower extremity arteries were detected by Philips HDll color Doppler ultrasound equipped with a linear array transducer (7–12 MHz variable frequency). The angle between the direction of the sound beam and the blood flow was less than 60°. The patients were positioned in supine position or prone position for all measurements. The femoral artery (FA), popliteal artery (PA), anterior tibial artery (ATA), and dorsalis pedis artery (DPA) were successively surveyed. Two-dimensional ultrasound was used to display the vessel long/short axis section images and detect plaque size, number, and vascular diameter. Color Doppler flow imaging (CDFI) was used to observe blood flow filling, vascular stenosis, and occlusion. The degree of artery stenosis was calculated by dividing the residual diameter (N) by the vessel diameter at a point distal to the stenosis where the normal vessel caliber was restored (D). The formula is presented as follows:

$$1 - {\text{N/D}} \times 1 0 0\,{ = }\,{\text{degree of stenosis}}$$

### Measurement of serum CML

Serum CML concentration of patients who underwent amputation was measured by CML ELISA Kit (Meixuan Co. Ltd, Shanghai, China) according to the manufacturer’s instructions. The mean minimum detectable concentration is 0.126  ng/mL. The antibodies in the CML kit are highly specific for CML adducts and do not exhibit cross reactivity to non-CML proteins in human plasma. The intra- and inter-assay coefficients of variation (CV) for CML are 5.2–7.4% and 4.7–15.2%, respectively.

### Disposal of human specimens

Anterior tibial artery samples were obtained from the foot of diabetic patients who underwent lower extremity amputation. After perfusion with cold phosphate buffer saline (PBS), each artery was cleaned to remove connective and adipose tissues and then divided longitudinally into three pieces. One piece was immediately frozen and stored at −80 °C until protein extraction for Western blot and ALP activity detection. One piece was used for calcium content detection, and the other piece was fixed in 10% neutral buffered formalin overnight and embedded in paraffin. From the paraffin blocks, 4 μm-thick serial sections were examined for hematoxylin–eosin (H&E) and von Kossa staining.

### Cell studies

RAW264.7 macrophages and A7r5 aortic VSMCs were cultured in DMEM/LOWGLUCOSE, supplemented with 10% fetal bovine serum (FBS), 4.0 mmol/L l-glutamine, 110 mg/L sodium pyruvate, 100 U/mL penicillin, and 100 μg/mL streptomycin. After RAW264.7 apoptosis induced by 50 μg/mL oxLDL for 48 h, the culture flasks were tapped to completely harvest the apoptotic bodies (ABs) as described previously [15]. The supernatant culture was centrifuged at 10,000×*g* (4 °C) for 20 min to remove cell debris. The resultant cell-free supernatant was re-centrifuged at 150,000×*g* (4 °C) for 1 h to obtain a pellet containing ABs. The pellet was washed three times with Hank’s Balanced Salt Solution (without calcium or magnesium). Protein content was determined using Bradford method.

The specific mechanism of CML in the transmission pathway of diabetic calcification cascade signal was also investigated using in vitro experiments. A7r5 VSMCs were divided into the following groups: control group (A7r5 medium supplemented with 80 μg/mL RAW264.7-derived-ABs plus 50 μg/mL oxLDL), CML group (A7r5 medium supplemented with 80 μg/mL RAW264.7-derived-ABs plus 50 μg/mL oxLDL, and 10 μmol/L CML), anti-RAGE group (A7r5 medium supplemented with 80 μg/mL RAW264.7-derived-ABs plus 50 μg/mL oxLDL, 10 μmol/L CML, and 100 μg/mL antibody against RAGE), NADPH oxidase inhibitor group (A7r5 medium supplemented with 80 μg/mL RAW264.7-derived-ABs plus 50 μg/mL oxLDL, 10 μmol/L CML, and 10 μmol/L DPI), p38MAPK inhibitor group (A7r5 medium supplemented with 80 μg/mL RAW264.7-derived-ABs plus 50 μg/mL oxLDL, 10 μmol/L CML, and SB203580), and anti-cbfα1 group (A7r5 medium supplemented with 80 μg/mL RAW264.7-derived-ABs plus 50 μg/mL oxLDL, 10 μmol/L CML, and 100 μg/mL antibody against cbfα1). Cells in each group were cultured for 7 days, and the medium was replaced every 2 days.

### von Kossa staining, calcium content assay, and ALP activity assay

#### von Kossa staining

The A7r5 cell climbing slices were fixed in 4% paraformaldehyde for 30 min and then washed twice with double-distilled water (ddH_2_O). The paraffin sections of the isolated anterior tibial artery were dewaxed and hydrated. Two slices were immersed in 1% silver nitrate for 30 min under intense sunbeam or ultraviolet light. The samples were then washed in distilled water to remove excess reagent, incubated with 5% sodium thiosulfate for 5 min, washed once in tap water and several times in distilled water, and finally counterstained with eosin (tissue) or neutral red (cell) for 10 min. After several washes, the slices were observed under an Olympus microscope.

#### Quantification of calcium content (or deposition)

Calcium content (or deposition) was determined as previously described [16]. Dried anterior tibial artery and A7r5 cells were decalcified with 0.6 N HCl for 24 h. The calcium content of HCl supernatant was determined colorimetrically through O-cresolphthalein complexone method. After the decalcification, the samples were washed three times with PBS and solubilized with 0.1 N NaOH-0.1% SDS. Protein content was measured using Bradford method. Calcium content of the samples was normalized with regard to protein content.

#### ALP activity assay

The ALP activity assay kit uses p-nitrophenyl phosphate (pNPP) as a phosphatase substrate, which turns yellow (λmax = 405 nm) when dephosphorylated by ALP. As previously described [17], the total proteins of tissues and cells were first extracted by centrifugation in RIPA lysis buffer. ALP activity was then measured colorimetrically. The results were normalized to the levels of total protein determined using Bradford method.

### Western blot analysis

Total proteins of anterior tibial artery and cells were initially extracted by centrifugation in RIPA lysis buffer. Equal amounts of protein samples were loaded into 10 or 12% SDS-PAGE gels and transferred onto a polyvinylidene difluoride (PVDF) membrane. The nonspecific proteins were blocked with 5% nonfat dried milk for 1 h. The membranes were incubated with the primary antibodies anti-CML (1:5000), anti-RAGE (1:500), anti-Nox4 (1:500), anti-P-p38 (1:500), anti-cbfα1 (1:500), anti-ALP (1:500), and anti-β-actin (1:1000) overnight at 4 °C as well as with secondary antibody (HRP-conjugated IgG) for 1 h. HRP-conjugated secondary antibodies were used in conjunction with an ECL chemiluminescence detection system. Protein expression was analyzed by Gel-Pro Analyzer 4 software and normalized to that of β-actin.

### Statistical analysis

Data were expressed as mean ± SD and analyzed by SPSS 13.0 software. For comparison between two variables, unpaired Student’s *t* test was used. Comparison among more than two groups was conducted using one-way analysis of variance (ANOVA), followed by post hoc LSD test. Pearson correlation analysis was used to detect the correlation between serum CML level and calcium content in the arterial wall. A two-tailed *P* < 0.05 was considered statistically significant.

## Results

### Baseline characteristics and laboratory data

Descriptive information regarding baseline characteristics and laboratory results is listed in Table 1. The evaluated indices including age, gender, smoking, diabetes duration, hypertension status, fasting plasma glucose, lipid profiles, BUN and SCr were not significantly different among the three groups. However, CML and HbA1c were significantly different among the three groups. These findings suggested a poor glycemic control for patients undergoing amputation before hospitalization.

**Table 1:** Baseline characteristics and laboratory data of the studied population

Variables	Mild stenosis	Moderate stenosis	Severe stenosis/occlusion
Males/females	9/6	8/7	8/7
Age (years)	65 ± 9	67 ± 8	71 ± 10
Diabetes duration (years)	10 ± 4	11 ± 5	12 ± 7
Hypertension (%)	67	67	73
Smoking (%)	33	40	33
FPG (mg/dL)	120.61 ± 13.53	130.87 ± 9.81	138.85 ± 15.80
HbA1c (%)	7.50 ± 1.62	9.31 ± 2.43*	12.33 ± 2.79*^,#^
Total cholesterol (mg/dL)	190.75 ± 32.58	188.51 ± 29.83	208.79 ± 48.13
Triglycerides (mg/dL)	172.51 ± 20.55	181.54 ± 18.73	192.81 ± 21.93
LDL-cholesterol (mg/dL)	142.61 ± 15.93	138.71 ± 12.89	158.71 ± 18.93
HDL-cholesterol (mg/dL)	40.51 ± 7.57	37.95 ± 6.17	41.21 ± 9.73
BUN (mmol/L)	6.03 ± 1.12	6.81 ± 0.73	6.92 ± 1.09
SCr (μmol/L)	103.51 ± 11.92	123.67 ± 8.83	131.54 ± 12.86
CML(ng/mL)	28.71 ± 4.81	36.80 ± 5.23*	57.66 ± 6.47*^,#^

Values are expressed as number (%) or mean ± SD, n = 15 for each group

*FPG* fasting plasma glucose, *HbA1c* glycated hemoglobin, *LDL* low density lipoprotein, *HDL* high density lipoprotein, *BUN* blood urea nitrogen, *SCr* serum creatinine, *CML* Nε-carboxymethyl-lysine

* *P* < 0.05, compared with the mild stenosis group

^#^ *P* < 0.05, compared with the moderate stenosis group

### Progression of atherosclerotic calcification in the foot of diabetic patients

**Fig. 1:**
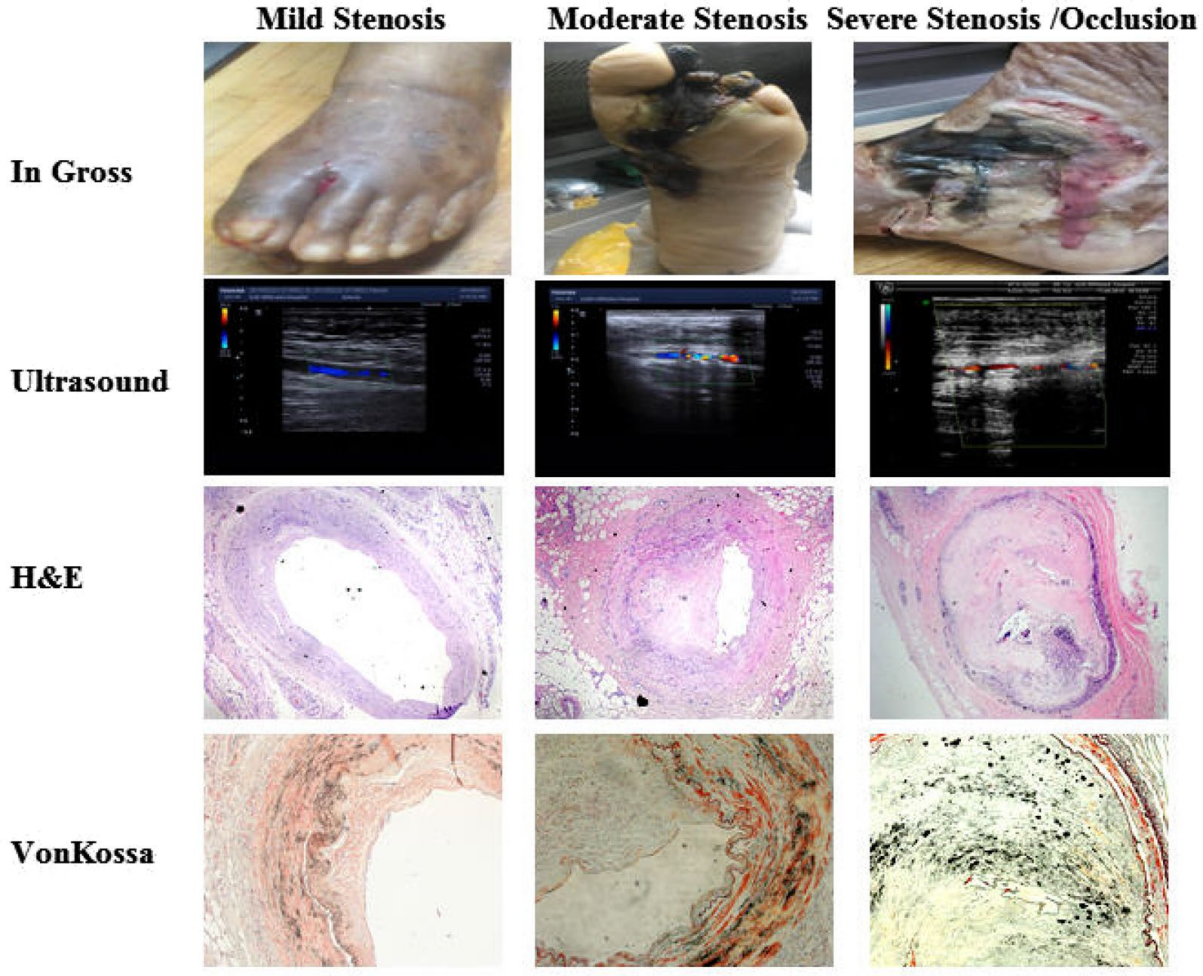
Progression of atherosclerotic calcification in diabetic foot patients. (1) In gross observation, different degrees of gangrene, swelling, skin ulcers, and infection in diabetic foot patients among the three groups were observed. (2) Ultrasound showed that echo-rich plaques (hard plaques) were the majority in the moderate stenosis/severe stenosis/occlusion group, whereas echolucent/heterogeneous plaques (soft/heterogeneous plaques) were predominant in the mild stenosis group. Furthermore, color blood flow in the severe stenosis/occlusion group became thin even in the absence of blood flow signal by CDFI. (3, 4) Representative photomicrographs of atherosclerotic lesions in anterior tibial artery cross-sections after H&E staining (×40) and von Kossa staining (black calcium particles) (×200). von Kossa staining revealed extensive calcification lesions in the intima and media of the anterior tibial artery. The extent and area of the calcium deposition in the intima became significantly more severe with disease progression. Spotty calcification was predominant in the atherosclerotic plaques of diabetic patients with amputation, whereas macrocalcification was almost invisible

In gross observation, different degrees of gangrene, swelling, skin ulcers, and infection in diabetic foot patients were observed among the three groups (Fig. 1). The extent/depth of the above lesions in moderate stenosis group and severe stenosis/occlusion group became more severe compared with the lesions in mild stenosis group. The results from two-dimensional ultrasound showed that echo-rich plaques (hard plaques) were mainly found in the moderate stenosis/severe stenosis/occlusion group, whereas echolucent/heterogeneous plaques (soft/heterogeneous plaques) were predominant in the mild stenosis group. Furthermore, color blood flow in the severe stenosis/occlusion group became thin even in the absence of blood flow signal by CDFI (Fig. 1).

**Fig. 2:**
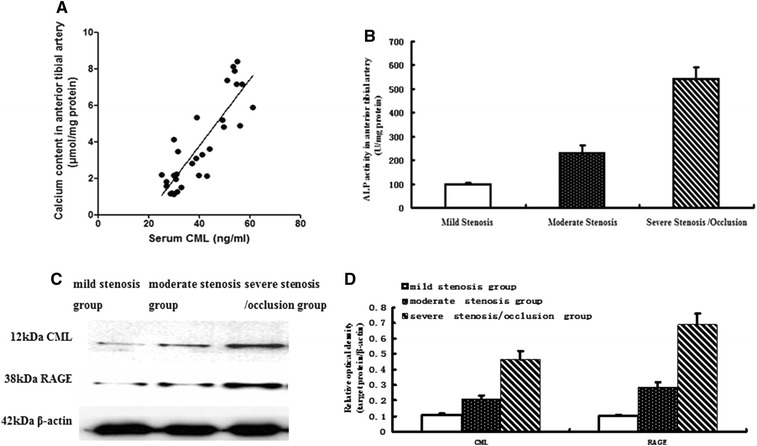
Changes in related indices in diabetic foot patients. **A** Pearson correlative analysis between serum CML level and calcium content in the anterior tibial arterial wall. The data revealed a significant positive correlation (R^2^ = 0.6141, *P* < 0.0001). **B** ALP activity in the anterior tibial artery. **C** Protein expression in the anterior tibial arterial wall of diabetic foot patients by Western blot. **D** Quantitative analysis of relative protein levels (target protein/β-actin) by densitometry. Values are expressed as mean ± SD, n = 15 for each group

In contrast to the lesions in the mild/moderate stenosis group, advanced atherosclerotic lesions with abundant cholesterol crystals and loss of integrity of the intern elastic lamina occurred in the severe stenosis/occlusion group (Fig. 1). von Kossa staining revealed extensive calcification lesions in the intima and media of the anterior tibial artery. Furthermore, the extent and area of the calcium deposition in the intima became significantly more severe with disease progression (Fig. 1). Interestingly, spotty calcification was predominant in the atherosclerotic plaques of amputated diabetic patients, whereas macrocalcification was almost invisible. Consistently, quantification analysis through O-cresolphthalein complexone method showed that calcium contents in the mild stenosis, moderate stenosis, and severe stenosis/occlusion groups are 1.60 ± 0.41, 3.23 ± 0.99, and 6.71 ± 1.38 μmol/mg, respectively (Fig. 2). The ALP activities in the three groups are 97.5 ± 9.12, 231.0 ± 31.5, and 541.7 ± 49.2 U/mg, respectively (Fig. 2). Our data suggested that atherosclerotic calcification in the foot of diabetic patients worsen with stenosis progression.

### Serum CML level and tissue CML/RAGE signal in the progression of atherosclerotic calcification

Serum CML level and tissue CML/RAGE signal in diabetic patients were detected by ELISA and Western blot analyses, respectively (Fig. 2). ELISA results showed that serum CML levels in the moderate stenosis group was increased by 1.28-fold (36.80 ± 5.23 versus 28.71 ± 4.81 ng/mL, *P* < 0.001) compared with those in the mild stenosis group. The index in the severe stenosis/occlusion group was increased by 1.57-fold (57.66 ± 6.47 versus 36.80 ± 5.23 ng/mL, *P* < 0.001) compared with that in the moderate stenosis group. Pearson correlation analysis revealed a significant positive correlation between serum CML level and calcium content in the arterial wall (R^2^ = 0.6141, *P* < 0.0001) (Fig. 2). Subsequently, semi-quantitative Western blot analysis indicated that the relative optical densities (target protein/β-actin) in the mild stenosis, moderate stenosis, and severe stenosis/occlusion groups are 0.106 ± 0.009, 0.211 ± 0.020, and 0.467 ± 0.054, respectively, in CML deposition as well as 0.103 ± 0.006, 0.287 ± 0.030, and 0.690 ± 0.071 in RAGE expression, respectively (Fig. 2). These results suggested that serum CML level and tissue CML/RAGE signal increased with progression of atherosclerotic calcification in diabetic patients.

### Signal pathway of CML-accelerated calcification progression

**Fig. 3:**
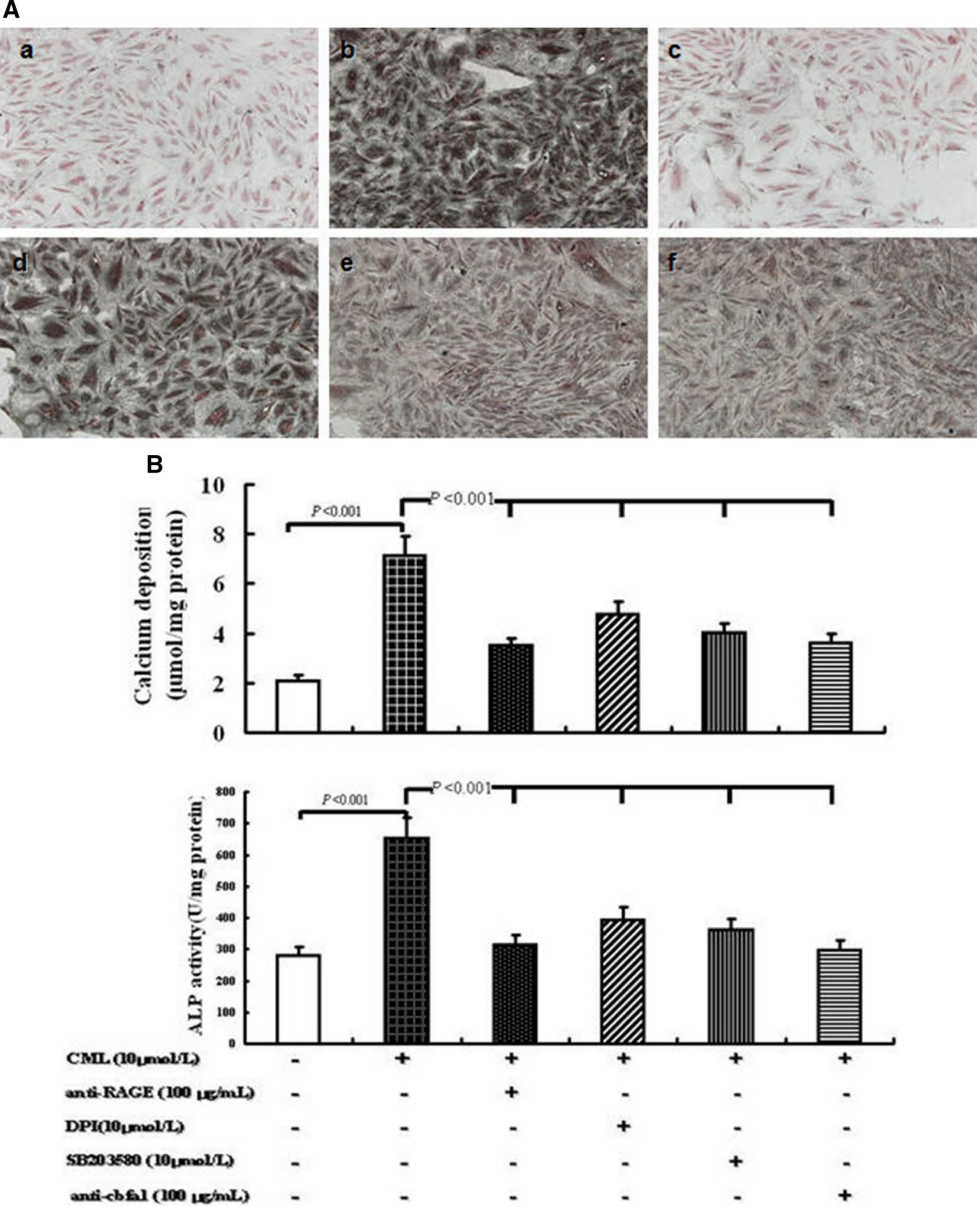
Effects of different treatments on VSMC calcification under high-lipid, apoptosis-coexisting conditions. **A** Morphology of calcified A7r5 cells was detected by von Kossa staining at the light microscopic level (×100). *a* Control group (A7r5 medium supplemented with 80 μg/mL RAW264.7-derived-ABs plus 50 μg/mL oxLDL); *b* CML group (A7r5 medium supplemented with 80 μg/mL RAW264.7-derived-ABs plus 50 μg/mL oxLDL and 10 μmol/L CML); *c* anti-RAGE group (A7r5 medium supplemented with 80 μg/mL RAW264.7-derived-ABs plus 50 μg/mL oxLDL, 10 μmol/L CML, and 100 μg/mL antibody against RAGE); *d* NADPH oxidase inhibitor group (A7r5 medium supplemented with 80 μg/mL RAW264.7-derived-ABs plus 50 μg/mL oxLDL, 10 μmol/L CML, and 10 μmol/L DPI); *e* p38MAPK inhibitor group (A7r5 medium supplemented with 80 μg/mL RAW264.7-derived-ABs plus 50 μg/mL oxLDL, 10 μmol/L CML, and SB203580); *f* anti-cbfα1 group (A7r5 medium supplemented with 80 μg/mL RAW264.7-derived-ABs plus 50 μg/mL oxLDL, 10 μmol/L CML, and 100 μg/mL antibody against cbfα1). Cells in each group were cultured for 7 days, and the medium was replaced every 2 days. **B** Calcium depositions were measured utilizing the O-cresolphthalein complexone method and normalized in accordance with the cellular protein content. The activity of ALP was analyzed by ALP activity assay kit. Cells were treated similar to **A**. Values are expressed as mean ± SD from the three independent experiments

**Fig. 4:**
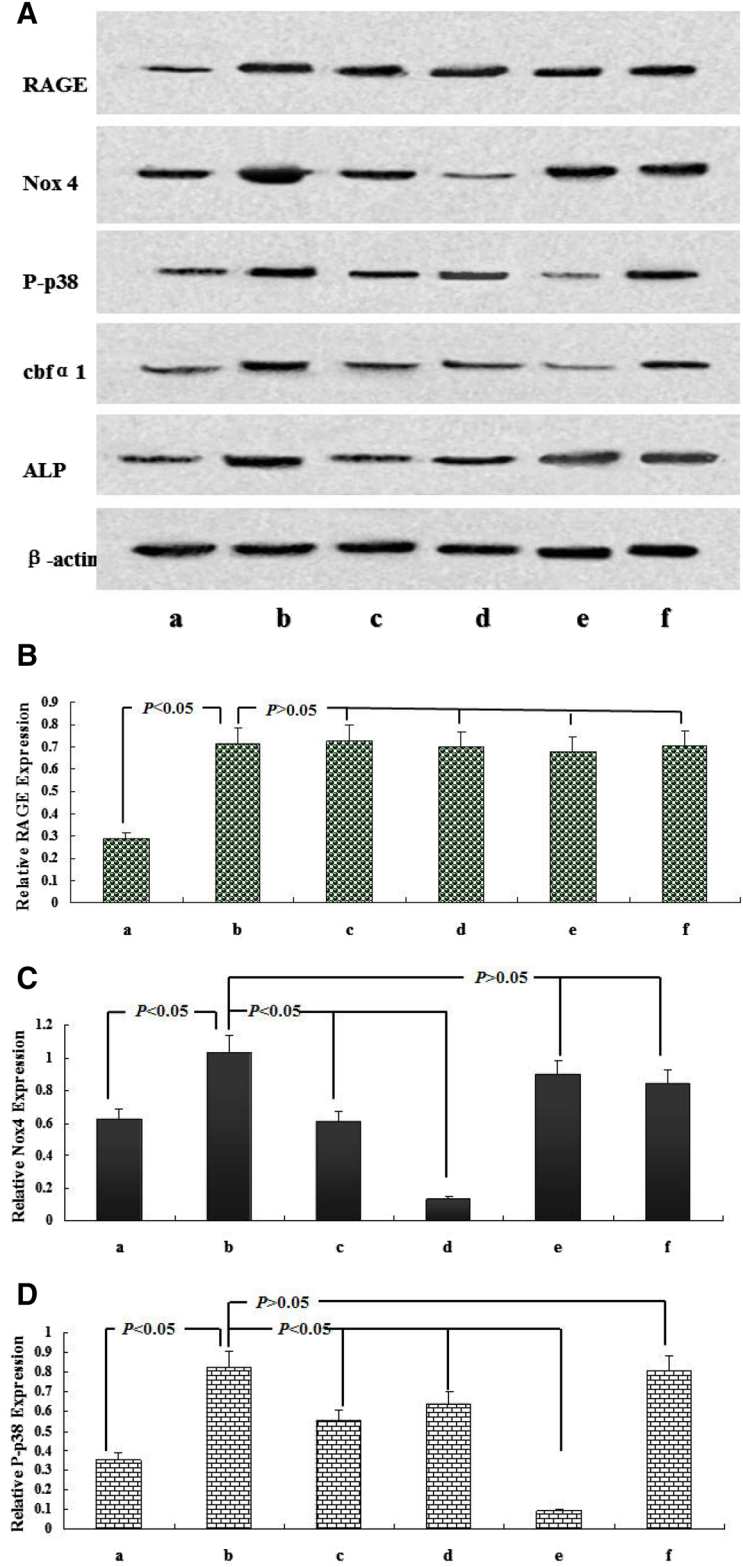
Molecular mechanism of CML-accelerated VSMC calcification under high-lipid, apoptosis-coexisting conditions. After the cells reached 80% confluence, A7r5 aortic smooth muscle cells were cultured in the presence of 50 μg/mL oxLDL and 80 μg/mL RAW264.7-derived-ABs with the indicated treatment for 7 days, and the medium was replaced every 2 days. **A** Presents the expression of related protein in treated A7r5 cells by Western blot. **B**–**D** present quantitative analysis of relative RAGE, Nox4 and P-p38 protein levels (target protein/β-actin) by densitometry. Values are expressed as mean ± SD from three independent experiments. *a* Control group (A7r5 medium supplemented with 80 μg/mL RAW264.7-derived-ABs plus 50 μg/mL oxLDL); *b* CML group (A7r5 medium supplemented with 80 μg/mL RAW264.7-derived-ABs plus 50 μg/mL oxLDL, and 10 μmol/L CML); *c* anti-RAGE group (A7r5 medium supplemented with 80 μg/mL RAW264.7-derived-ABs plus 50 μg/mL oxLDL, 10 μmol/L CML, and 100 μg/mL antibody against RAGE); *d* NADPH oxidase inhibitor group (A7r5 medium supplemented with 80 μg/mL RAW264.7-derived-ABs plus 50 μg/mL oxLDL, 10 μmol/L CML, and 10 μmol/L DPI); *e* p38MAPK inhibitor group (A7r5 medium supplemented with 80 μg/mL RAW264.7-derived-ABs plus 50 μg/mL oxLDL, 10 μmol/L CML, and SB203580); *f* anti-cbfα1 group (A7r5 medium supplemented with 80 μg/mL RAW264.7-derived-ABs plus 50 μg/mL oxLDL, 10 μmol/L CML, and 100 μg/mL antibody against cbfα1). Values are expressed as mean ± SD from three independent experiments

**Fig. 5:**
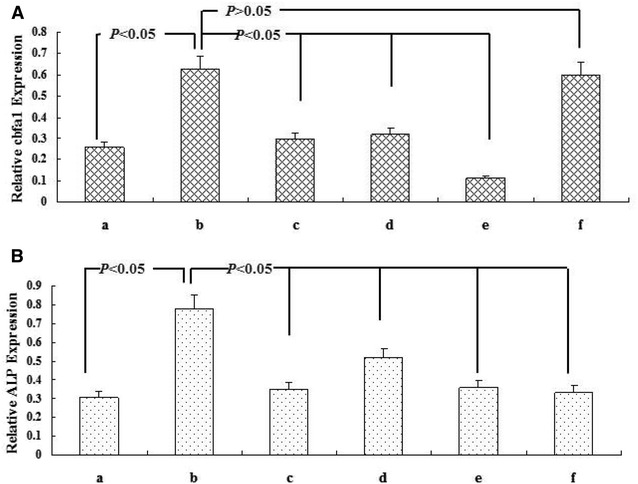
Molecular mechanism of CML-accelerated VSMC calcification under high-lipid, apoptosis-coexisting conditions. After the cells reached 80% confluence, A7r5 aortic smooth muscle cells were cultured in the presence of 50 μg/mL oxLDL and 80 μg/mL RAW264.7-derived-ABs with the indicated treatment for 7 days, and the medium was replaced every 2 days. **A**, **B** present quantitative analysis of relative cbfα1, and ALP protein levels (target protein/β-actin) by densitometry. Values are expressed as mean ± SD from three independent experiments. *a* Control group (A7r5 medium supplemented with 80 μg/mL RAW264.7-derived-ABs plus 50 μg/mL oxLDL); *b* CML group (A7r5 medium supplemented with 80 μg/mL RAW264.7-derived-ABs plus 50 μg/mL oxLDL, and 10 μmol/L CML); *c* anti-RAGE group (A7r5 medium supplemented with 80 μg/mL RAW264.7-derived-ABs plus 50 μg/mL oxLDL, 10 μmol/L CML, and 100 μg/mL antibody against RAGE); *d* NADPH oxidase inhibitor group (A7r5 medium supplemented with 80 μg/mL RAW264.7-derived-ABs plus 50 μg/mL oxLDL, 10 μmol/L CML, and 10 μmol/L DPI); *e* p38MAPK inhibitor group (A7r5 medium supplemented with 80 μg/mL RAW264.7-derived-ABs plus 50 μg/mL oxLDL, 10 μmol/L CML, and SB203580); *f* anti-cbfα1 group (A7r5 medium supplemented with 80 μg/mL RAW264.7-derived-ABs plus 50 μg/mL oxLDL, 10 μmol/L CML, and 100 μg/mL antibody against cbfα1). Values are expressed as mean ± SD from three independent experiments

Studies on diabetic patients and apoE^−/−^ mice [10] indicated the important role of CML in atherosclerotic calcification. However, the mechanism of CML in the transmission pathway of diabetic calcification cascade signal remains unclear. In our subsequent study, the related pathway was blocked by anti-RAGE antibody, NADPH oxidase inhibitor DPI, p38MAPK inhibitor SB203580, and anti-cbfa1 antibody in a step-wise manner to observe changes in calcium deposition and related molecular signals. Calcium deposition was assessed by von Kossa staining and O-cresolphthalein complexone method. ALP activity and related protein expression were examined by ALP kit and semi-quantitative Western blot analysis (Figs. 3, 4, 5).

Compared with the indices in the CML-oxLDL-Abs group, the anti-RAGE blocking group under high-lipid, apoptosis-coexisting conditions induced the following changes. ALP activity was inhibited by 51.8% (314.5 ± 28.7 versus 652.8 ± 63.3 U/mg, *P* < 0.001). Intercellular calcium deposition was reduced by 50.6% (3.52 ± 0.29 versus 7.12 ± 0.81 μmol/mg, *P* < 0.001). The expression levels of Nox4, P-p38, cbfα1, and ALP were decreased by 39.6, 32.5, 53.0, and 55.2%, respectively.

When pretreated with NADPH oxidase inhibitor DPI, ALP activity and Intercellular calcium deposition were reduced by 39.7% (393.6 ± 31.2 versus 652.8 ± 63.3 U/mg, *P* < 0.001) and 33.0% (4.77 ± 0.53 versus 7.12 ± 0.81 μmol/mg, *P* < 0.001), respectively, comparing with CML-oxLDL-Abs group. Meanwhile, the expression levels of Nox4, P-p38, cbfα1, and ALP in western blot were decreased by 86.8, 22.4, 49.4, and 33.2%, respectively, but there were no significant changes in the expression of RAGE.

Subsequently, the treatment with p38MAPK inhibitor SB203580 induced similar changes with DPI: ALP activity was inhibited by 44.6% (361.8 ± 33.3 versus 652.8 ± 63.3 U/mg, *P* < 0.001) and intercellular calcium deposition was reduced by 43.7% (4.01 ± 0.39 versus 7.12 ± 0.81 μmol/mg, *P* < 0.001). However, the expression levels of P-p38, cbfα1, and ALP were downregulated by 88.7, 82.2, and 53.8%, respectively. Interestingly, no significant changes in the expression of RAGE and Nox4 were observed.

Moreover, compared with CML-oxLDL-Abs group, the treatment with anti-cbfa1 antibody could inhibite ALP activity by 54.5% (297.2 ± 26.5 versus 652.8 ± 63.3 U/mg, *P* < 0.001) and reduced intercellular calcium deposition by 49.0% (3.63 ± 0.33 versus 7.12 ± 0.81 μmol/mg, *P* < 0.001). And importantly, anti-cbfa1 antibody reduced the expression level of ALP by 57.1%. However, it had no significant effects on the expression of RAGE, Nox4, P-p38, and cbfα1. Hence, our data above suggested that CML may play a key role in atherosclerotic calcification mainly through CML/RAGE-ROS-p38MAPK-cbfα1-ALP pathway.

## Discussion

Vascular calcification is a significant predictor of coronary heart disease events, stroke, and lower-limb amputation [18, 19]. Compared with diabetics without vascular calcification, diabetics with vascular calcification exhibited a 1.5-fold increase in mortality, a 1.6-fold increase in coronary artery disease, a 2.4-fold increase in proteinuria, a 1.7-fold increase in retinopathy, and a 5.5-fold increase in amputation [20]. Among various types of vascular calcification, spotty calcification (namely microcalcification) in atherosclerotic plaques is the most important because they promote plaque instability and acute cardiocerebrovascular events [19, 21]. Our data showed that spotty calcification was widely distributed in the atherosclerotic plaques of amputated diabetic patients, which may result in the occurrence of diabetic complications. The results of molecular biological analysis suggested that CML deposition and RAGE expression in the arterial wall were upregulated with the development of atherosclerotic calcification. Pearson correlative analysis revealed a significant positive correlation between serum CML level and calcium content in the arterial wall. Furthermore, blocking treatments with anti-RAGE antibody could significantly inhibit the osteogenic differentiation of A7r5 smooth muscle cells induced by CML under high-lipid, apoptosis-coexisting conditions. Our previous and present studies [10] suggested that CML/RAGE signal could promote the development of diabetic calcification within atherosclerotic plaques. Other studies also suggested that the formation of microcalcification and increase in plaque instability could be specifically induced by AGEs and RAGE signal [22, 23]. However, many inconsistencies were observed in existing studies particularly on the precise pathway through which calcification cascade signals are transmitted. Thus, the related mechanism must be further elaborated.

The interaction between AGEs and RAGE enhances intracellular oxidative stress and promotes the production of reactive oxygen species (ROS) [24, 25]. ROS, a by-product of the body’s metabolism, regulates the structure and function of blood vessel wall and is an important physiological/pathological regulatory mediator of intracellular signal cascades [26]. Among the various source of ROS generation such as NADPH oxidases, mitochondrial electron transport xanthine oxidase, peroxisomes, cytochrome *P*-450 and NO synthases (NOS), the Nox family of NADPH oxidases including seven different enzymes is the principal source [27–29]. Their wide functions depend not only on the Nox isoform but also on cell types. Evidences have suggested NADPH oxidase activity in vascular smooth muscles mainly relied on Nox-1, -4, and -5 expressions [30, 31]. Furthermore, Nox4-derived ROS is involved in the regulation of VSMC phenotype differentiation. Once Nox4 is blocked by siRNA, the specific markers of VSMC could either be lost or downregulated [32, 33]. Given that the osteogenic differentiation of VSMC plays a key role in vascular calcification, we subsequently observed changes of Nox4 in the CML/RAGE signal pathway.

Early studies revealed that AGEs could mediate the activation of p38MAPK signals through ROS [34].In addition, cbfα1 serves as a key transcription factor in osteogenic differentiation, closely linked with vascular calcification [35]. Thus, in order to determine whether CML, the major active ingredient of AGEs, could induce the cascade of calcification signal through ROS and p38MAPK in VSMC, the NADPH oxidase inhibitor DPI, p38MAPK inhibitor SB203580 and anti-cbfα1 antibody were utilized in present study to observe the mechanism.

Firstly, in the model of CML-accelerated VSMC calcification under high-lipid, apoptosis-coexisting conditions, anti-RAGE antibody, NADPH oxidase inhibitor DPI, p38MAPK inhibitor SB203580 and anti-cbfα1 antibody significantly inhibited ALP activity and intercellular calcium deposition, but the specific signal stream was unclear. Then the expressions of related signal protein were further established. The expression levels of Nox4, P-p38, cbfα1 and ALP were significantly decreased when pretreated with NADPH oxidase inhibitor DPI, but no significant changes in the expression of RAGE, indicating that CML/RAGE signal was the upstream signals of Nox-derived ROS, p38MAPK, cbfα1 and ALP. Subsequently, our data showed that the treatment with p38MAPK inhibitor SB203580 downregulated the expression of P-p38, cbfα1 and ALP. However, no significant changes in the expression of RAGE and Nox4 were observed, suggesting p38MAPK, cbfα1 and ALP were the downstream molecules of Nox-derived ROS. Now that the expression of cbfα1 could be significantly inhibited at different extents by DPI and SB203580, respectively, blocking experiment with anti-cbfα1 antibody was performed. The ALP expression was significantly reduced by anti-cbfα1 antibody. However, no significant changes in the expression of RAGE, Nox4, P-p38, and cbfα1 were observed, which shed light on that cbfα1 was the upstream molecule of ALP, but downstream molecule of p38MAPK. Considering these results, we conclude that CML/RAGE–ROS-p38MAPK-cbfα1- ALP calcification may be the most important cascade of CML/RAGE in diabetic calcification.

## Conclusion

In conclusion, these findings suggest that spotty calcification was predominant in the atherosclerotic plaques of amputated diabetic patients and that CML/RAGE signal may induce the calcification cascade in diabetes via ROS-p38MAPK. On the basis of the target signal of the calcification cascade, related drug development and subsequent optimization of an intervention strategy would provide new opportunities for clinical treatment of chronic vascular complications of diabetes.

## Abbreviations

AGEs: advanced glycation end-products
CML: Nε-carboxymethyl-lysine
RAGE: receptor for advanced glycation end products
Nox4: NADPH oxidase 4
cbfα1: core-binding factor α1
ALP: alkaline phosphatase
ROS: reactive oxygen species
VSMCs: vascular smooth muscle cells
FPG: fasting plasma glucose
HbA1c: glycated hemoglobin
LDL: low-density lipoprotein
HDL: high-density lipoprotein
BUN: blood urea nitrogen
SCr: serum creatinine
CDFI: color Doppler flow imaging
PBS: phosphate buffer saline
FBS: fetal bovine serum
Abs: apoptotic bodies
PVDF: polyvinylidene difluoride
NOS: NO synthases

## References

[1] International Diabetes Federation (2014). IDF diabetes atlas 6th edition revision 2014.

[2] Lilly SM, Qasim AN, Mulvey CK (2013). Non-compressible arterial disease and the risk of coronary calcification in type-2 diabetes. Atherosclerosis.

[3] Patsch JM, Zulliger MA, Vilayphou N (2014). Quantification of lower leg arterial calcifications by high-resolution peripheral quantitative computed tomography. Bone.

[4] Bild DE, Detrano R, Peterson D (2005). Ethnic differences in coronary calcification: the Multi-Ethnic Study of Atherosclerosis (MESA). Circulation.

[5] Detrano R, Guerci AD, Carr JJ (2008). Coronary calcium as a predictor of coronary events in four racial or ethnic groups. N Engl J Med.

[6] Shah S, Bellam N, Leipsic J (2014). Prognostic significance of calcified plaque among symptomatic patients with nonobstructive coronary artery disease. J Nucl Cardiol..

[7] Kataoka Y, Wolski K, Uno K (2012). Spotty calcification as a marker of accelerated progression of coronary atherosclerosis: insights from serial intravascular ultrasound. J Am Coll Cardiol.

[8] Allison MA, Hsi S, Wassel CL (2012). Calcified atherosclerosis in different vascular beds and the risk of mortality. Arterioscler Thromb Vasc Biol.

[9] McCarty MF, DiNicolantonio JJ (2014). The molecular biology and pathophysiology of vascular calcification. Postgrad Med.

[10] Wang Z, Jiang Y, Liu N (2012). Advanced glycation end-product N epsilon-carboxymethyl-Lysine accelerates progression of atherosclerotic calcification in diabetes. Atherosclerosis.

[11] Yamagishi S, Fukami K, Matsui T (2015). Evaluation of tissue accumulation levels of advanced glycation end products by skin autofluorescence: a novel marker of vascular complications in high-risk patients for cardiovascular disease. Int J Cardiol.

[12] Nowotny K, Jung T, Höhn A (2015). Advanced glycation end products and oxidative stress in type 2 diabetes mellitus. Biomolecules.

[13] Fukushima Y, Daida H, Morimoto T (2013). Relationship between advanced glycation end products and plaque progression in patients with acute coronary syndrome: the JAPAN-ACS sub-study. Cardiovasc Diabetol.

[14] Kay AM, Simpson CL, Stewart JA (2016). The role of AGE/RAGE signaling in diabetes-mediated vascular calcification. J Diabetes Res.

[15] Hashimoto S, Ochs RL, Rosen F (1998). Chondrocyte-derived apoptotic bodies and calcification of articular cartilage. Proc Natl Acad Sci USA.

[16] Ren X, Shao H, Wei Q (2009). Advanced glycation end-products enhance calcification in vascular smooth muscle cells. J Int Med Res.

[17] Duan X, Zhou Y, Teng X (2009). Endoplasmic reticulum stress-mediated apoptosis is activated in vascular calcification. Biochem Biophys Res Commun.

[18] Nguyen N, Naik V, Speer MY (2013). Diabetes mellitus accelerates cartilaginous metaplasia and calcification in atherosclerotic vessels of LDLr mutant mice. Cardiovasc Pathol.

[19] Hutcheson JD, Maldonado N, Aikawa E (2014). Small entities with large impact: microcalcifications and atherosclerotic plaque vulnerability. Curr Opin Lipidol.

[20] Everhart JE, Pettitt DJ, Knowler WC (1988). Medial arterial calcification and its association with mortality and complications of diabetes. Diabetologia.

[21] Baumann S, Renker M, Meinel FG (2015). Computed tomography imaging of coronary artery plaque: characterization and prognosis. Radiol Clin North Am.

[22] Pugliese G, Iacobini C, Blasetti FC (2015). The dark and bright side of atherosclerotic calcification. Atherosclerosis..

[23] Menini S, Iacobini C, Ricci C (2013). The galectin-3/RAGE dyad modulates vascular osteogenesis in atherosclerosis. Cardiovasc Res.

[24] Brodeur MR, Bouvet C, Bouchard S (2014). Reduction of advanced-glycation end products levels and inhibition of RAGE signaling decreases rat vascular calcification induced by diabetes. PLoS ONE.

[25] Basta G, Lazzerini G, Del Turco S (2005). At least 2 distinct pathways generating reactive oxygen species mediate vascular cell adhesion molecule-1 induction by advanced glycation end products. Arterioscler Thromb Vasc Biol.

[26] Park JG, Oh GT (2011). The role of peroxidases in the pathogenesis of atherosclerosis. BMB Rep.

[27] Kim JA, Neupane GP, Lee ES (2011). NADPH oxidase inhibitors: a patent review. Expert Opin Ther Pat.

[28] Montezano AC, Burger D, Ceravolo GS (2011). Novel Nox homologues in the vasculature: focusing on Nox4 and Nox5. Clin Sci (Lond).

[29] Rivera J, Sobey CG, Walduck AK (2010). Nox isoforms in vascular pathophysiology: insights from transgenic and knockout mouse models. Redox Rep.

[30] Bedard K, Krause KH (2007). The NOX family of ROS-generating NADPH oxidases: physiology and pathophysiology. Physiol Rev.

[31] Kim M, Han CH, Lee MY (2014). NADPH oxidase and the cardiovascular toxicity associated with smoking. Toxicol Res.

[32] Clempus RE, Sorescu D, Dikalova AE (2007). Nox4 is required for maintenance of the differentiated vascular smooth muscle cell phenotype. Arterioscler Thromb Vasc Biol.

[33] Deliri H, McNamara CA (2007). Nox 4 regulation of vascular smooth muscle cell differentiation marker gene expression. Arterioscler Thromb Vasc Biol.

[34] Yang K, Wang XQ, He YS (2010). Advanced glycation end products induce chemokine/cytokine production via activation of p38 pathway and inhibit proliferation and migration of bone marrow mesenchymal stem cells. Cardiovasc Diabetol.

[35] Bai Y, Zhang J, Xu J (2015). Magnesium prevents β-glycerophosphate-induced calcification in rat aortic vascular smooth muscle cells. Biomed Rep.

